# Association Between Calcium and Protein Content of Breast Milk and Severe Early Childhood Caries (S-ECC) in Toddlers

**DOI:** 10.34172/joddd.44503

**Published:** 2026-03-30

**Authors:** Hamidreza Poureslami, Salehe Sabouri, Seerat Abbas Rajput, Milad Mollaali, Reyhaneh Aftabi, Parnian Poureslami, Abolfazl Shiri Varnamkhasti, Mohammad Reza Arabnezhad

**Affiliations:** ^1^Department of Pediatric Dentistry, Dental Faculty, Kerman University of Medical Sciences, Kerman, Iran; ^2^Pharmaceutics Research Center, Institute of Pharmaceutical Sciences, Kerman University of Medical Sciences, Kerman, Iran; ^3^Department of Pharmaceutical Biotechnology, Faculty of Pharmacy, Kerman University of Medical Sciences, Kerman, Iran; ^4^Department of Biology, Faculty of Science, University of Sistan and Baluchestan, Zahedan, Iran

**Keywords:** Breastfeeding, Calcium, Child, Child oral health, Dental caries, Proteins

## Abstract

**Introduction:**

Severe early childhood caries (S-ECC) is a special type of dental decay common among toddlers and young children. This study explored the association between S-ECC and calcium and protein levels in mothers’ breast milk in toddlers aged 12‒24 months.

**Methods:**

This cross-sectional descriptive-analytical study included 33 toddlers aged 12‒24 months, with or without S-ECC. Participants were recruited from mothers who brought their toddlers to healthcare clinics in Kerman City, Iran, for routine check-ups and vaccinations. The inclusion criteria for mothers were ages 18‒45 years during pregnancy and delivery at 37 weeks of gestation or later. Breast milk samples were obtained from the mothers of 17 toddlers with S-ECC and from the mothers of 16 toddlers with healthy teeth. The protein and calcium levels in the samples were measured using the Bradford assay and Flame Atomic Absorption Spectrophotometry assay, respectively.

**Results:**

The study found a significantly higher mean protein concentration but no significant difference in calcium concentration in breast milk from mothers whose children were caries-free compared with those whose children had caries. Also, the refined regression model predicted a significant inverse association between protein concentration and dental caries (OR=0.171). Additionally, boys exhibited 14.8 times greater odds of caries compared to girls (OR=14.818).

**Conclusion:**

It can be suggested that increased protein intake from breastfeeding in toddlers younger than 24 months, along with being female, is linked to a reduced risk of S-ECC.

## Introduction

 Oral health is an integral part of overall health. Despite increased awareness among the general public and healthcare providers, early childhood caries (ECC) remains an important public health concern. While caries prevalence in permanent teeth has declined, it has remained constant or even increased in deciduous teeth in certain populations.^1^ ECC has reached epidemic proportions in communities with low socioeconomic status and in underserved areas. ECC is considered one of the most serious dental problems in toddlers and young children. Various definitions have been proposed to describe this condition. The American Academy of Pediatric Dentistry (AAPD) defines ECC as the presence of at least one primary tooth with caries, whether it is cavitated or not, missing due to tooth decay, or filled, in children who are 71 months old or younger. However, when children under the age of three display any caries on a smooth surface, it is considered severe early childhood caries (S-ECC).^1,2^

 There is a general agreement that individuals affected by ECC predominantly belong to the socioeconomically disadvantaged groups.^2^ The affected children often exhibit extensive colonization by *Streptococcus mutans* and display poor nutritional strategies.^1^ The routine consumption of sugar-based substances and night-time formula feeding with sweetened liquids are examples of inappropriate dietary practices that have been consistently linked with ECC in numerous investigations.^2^ In light of these suggestions, the American Academy of Pediatric Dentistry (AAPD), in the first clause of its policy on promoting appropriate nutritional practices and discouraging harmful feeding behaviors, states that night-time and on-demand breastfeeding need to be stopped following the eruption of the first primary tooth.^3^ There is no evidence linking breastfeeding until the age of one to an increased risk of dental caries; in comparison to formula feeding, it might offer protective benefits. However, recent studies indicate that the risk of dental caries increases among infants who are breastfed for more than 12 months. Furthermore, an obvious relationship exists between the severity of caries in a deciduous tooth and extended breastfeeding beyond 2 years.^4^ The American Academy of Pediatrics (AAP) stated that breastfeeding should persist for a minimum of one year and may be prolonged at the discretion of the child and mother. No specific age limit for breastfeeding is mentioned. Similarly, the World Health Organization (WHO) advocates continued breastfeeding until the child reaches 24 months of age.^5^ Although ECC is not life-threatening, it can lead to an adverse impact on children’s overall health and development.^6^

 Breast milk’s proteins are divided into three distinct types: caseins, milk fat globule membrane (MFGM), and whey proteins, which include secretory immunoglobulin A (s-IgA), lactoferrin, and alpha-lactalbumin. The concentrations of casein and whey proteins change significantly throughout the breastfeeding period. During the initial stages of lactation, the amounts of aqueous proteins are extremely substantial, whereas casein is almost indistinguishable. As the infant grows, the synthesis of casein increases and consequently, the concentration of casein rises, partially as a result of the mother’s hormonal fluctuations. Eventually, the ratio reaches approximately 80% casein and 20% whey protein. The amino acid content of breast milk changes as the infant develops, in part due to variations in whey protein and casein profiles.^7^

 Evaluation of actual protein content has stated levels of 14‒16 grams per liter (g/L) in the initial phases of lactation: 7‒8 g/L at six months and 8‒10 g/L at three to four months. Nevertheless, the quantity of functional amino acids in infants is not precisely determined by their actual protein intake, as some milk proteins may remain undigested in their stools. For example, lactoferrin and s-IgA are found in relatively high amounts in the stools of breastfed infants.^8^ Immunoglobulins (particularly s-IgA), lactoferrin, β-casein, α-lactalbumin, and lysozyme are among the most significant bioactive proteins contained within human milk.^9,10^ Caseins function as antimicrobial peptides, probably by disrupting or inhibiting cell division.^11^ Additionally, caseins can prohibit the adhesion of *S. mutans* to the biofilm of dental enamel or hydroxyapatite.^4^ The highest ability to inhibit bacterial adhesion is observed in caseins, followed by lactoferrin and IgA, while this ability is not seen in albumins and lysozymes.^12^ Two studies have shown the protein’s inhibitory effects on the adhesion of four subtypes of *S. mutans* and two subtypes of *S. sobrinus.*^13,14^ The reduction in adhesion triggered by caseins decreases *S. mutans* counts in dental enamel biofilms, thereby reducing plaque pathogenicity. As a result, it reduces the risk of enamel demineralization and the initiation of caries locally. Research has shown that IgA present in breast milk plays a protective role against the replication of *S. mutans*, a bacterium associated with dental caries. Frequent and sustained contact may decrease plaque pH, leading to enamel demineralization. Research suggests that extended breastfeeding, such as 12, 18, or 24 months, may be associated with an increased risk of caries, and it encourages considering adjustments to the number and duration of night-time breastfeeding.^15^

 Calcium is the second most important messenger in cellular signaling pathways. At the same time, phosphorus, in addition to its abundance in bones (which also requires magnesium), is a key element of nucleic acids and cell membranes. A calcium imbalance can significantly affect tooth structure during pregnancy and childhood. Diets that lack calcium or have a low calcium-to-phosphorus ratio (1:3) can cause hypo-mineralization defects in enamel and dentin, microdontia, and delayed eruption. Additionally, calcium and vitamin D deficiencies reduce the overall tooth weight.^16^ Calcium in breast milk significantly enhances the buffering capacity of saliva, thereby preventing demineralization and promoting remineralization in teeth. However, if another carbohydrate source, such as formula milk, is introduced into the child’s diet, there is a risk of shifting the balance towards lower pH and demineralization. Consequently, as the protein content in breast milk decreases after the first year, the risk of caries increases with prolonged breastfeeding.^17^ No study was found that examined the association between the manifestation of S-ECC and the amounts of calcium and protein. Therefore, this research aims to determine the association between S-ECC and calcium and protein levels in the breast milk of mothers with toddlers aged 12‒24 months in Kerman City, Iran, in 2025.

## Methods

 This research involved 33 toddlers aged 12‒24 months, both with and without S-ECC, along with their mothers. The Ethics Committee of Kerman University of Medical Sciences granted ethical approval for this research (IR.KMU.REC.1403.358). Also, informed consent was obtained from mothers. The sample size was determined using the formula N = P*q*z^2^/d, based on similar studies, and a simple sampling method.^18^ The participants were recruited from mothers who brought their toddlers to healthcare clinics in Kerman City, Iran, for routine check-ups and vaccinations. The inclusion criteria for mothers were an age range of 18‒45 years and a pregnancy lasting at least 37 weeks. The ethnic origin of all the mothers and toddlers in the study was Iranian. Mother‒toddler dyads were selected based on specific inclusion criteria related to hygiene and diet. Participating toddlers were required to have had their teeth cleaned by their mothers using a finger toothbrush since the onset of tooth eruption (approximately 6–7 months of age). The children’s daily nutrition was restricted to breast milk and complementary (semi-solid) foods. Also, toddlers who consumed snacks were excluded. Mothers were excluded if they had a history of diabetes, smoking, alcohol consumption, or if they fed their babies formula. These exclusion criteria were applied because the above-mentioned factors could have a confounding effect on our sample by affecting the composition of the mother’s breast milk. Experimental evaluation of the toddler’s teeth was executed using a disposable mouth mirror and under sufficient light after cleaning the surface of the upper anterior teeth with sterile gauze. Upon observing any signs of caries, including decalcification with or without discoloration, cavitation, on the labial surfaces of the maxillary incisor teeth, a diagnosis of S-ECC was made. Additionally, the International Caries Detection and Assessment System (ICDAS) criteria were used to diagnose S-ECC in these toddlers (1). The examiner’s calibration was performed by a pediatric dentistry professor who examined 20 toddlers with S-ECC, and the process continued until the professor and the examiner reached 70% agreement in diagnosing S-ECC. Seventeen breast milk samples were collected from mothers of toddlers with S-ECC, and sixteen samples from mothers whose toddlers had healthy teeth. Breast milk samples were collected by the mothers at their convenience, at any time from early morning through the day, and subsequently delivered in sterile 50 mL containers. The samples were frozen at -20°C, and after all samples were collected, protein and calcium analyses were performed.

 To measure protein content, the method described by Ramiro-Cortijo et al.^19^ was used with some modifications. Briefly, 1 mL of milk was centrifuged 3 times for 10 minutes at 2000 g and 4°C to separate the milk fat. The Bradford assay for protein quantification was performed by adding 900 µL of pure distilled water to 100 µL of the centrifuged milk. Then 10 µL of this mixture was combined with 200 µL of Bradford reagent, stirred for 1 minute, and its absorbance was determined in a microplate reader (Synergy HT Multimode; BioTek instruments, USA) at 595 nm. Bovine serum albumin (BSA; Sigma-Aldrich, USA) was used to plot the standard curve. Total protein levels in milk samples were expressed as g/L. Calcium concentration was assayed by Flame Atomic Absorption Spectrophotometry (FAAS) based on Goc et al.^20^ Briefly, 1 mL of milk samples was mixed with 2 mL of HNO_3_-HClO_4_ (at the ratio of 4:1). After heating for 240 minutes at 120°C in a thermostat-controlled digestion block, the samples were cooled and brought to 5 mL with demineralized water. The calcium concentration was calculated in mg/L. Calcium carbonate (CaCO_3_) was used to plot the standard curve, with calculations based on the amount of calcium.

 Descriptive statistics were used for data analysis with SPSS 27 (IBM Corp., Armonk, NY, USA). Additionally, the means and standard deviations (SD) were computed to assess calcium and protein levels. To compare calcium and protein levels in breast milk from mothers of toddlers with S-ECC and those of healthy toddlers, an independent t-test was conducted. Finally, binary logistic regression using the Enter method was used to construct models for predicting dental caries in toddlers. The threshold for statistical significance was established at *P* < 0.05.

## Results

 The study population comprised 33 subjects. The average age of the mothers was 28.45 ± 3.3 years, and the median age of the toddlers was 16 months. It is important to note that the maximum and minimum ages of the mothers in the sample were 34 and 24 years, respectively, while the maximum and minimum ages of the toddlers were 20 and 12 months, respectively. According to the results, the majority of the mothers in the sample were > 25 years old, while the majority of the toddlers were > 18 months old (Table 1). The gender distribution of toddlers in the study was 17 boys (51.5%) and 16 girls (48.5%).

**Table 1:** Age of mothers and their toddlers participating in the study

	**N**	**Mean**	**SD**	**Mode**	**Median**	**Max**	**Min**
Mothers’ age (years)	33	28.45	3.3	25	27	34	24
Toddlers’ age (months)	33	16.27	2.06	18	16	20	12

SD = standard deviation, Max = minimum, Min = maximum

 The independent t-test (Table 2) showed no statistically significant difference in calcium concentrations between toddlers with and without dental caries (t = -0.865, *P* = 0.39, Cohen’s d = -0.301); however, the protein concentration was markedly elevated in the caries-free group in contrast to the caries group (t = 2.213, *P* = 0.04, Cohen’s d = 0.771).

**Table 2:** Calcium and protein concentrations in the breast milk samples in the two groups

**Compounds**	**Caries status**	**N**	**Mean**	**SD**	**t**	***P*** **-value**^¶^	**Cohen’s d**^†^
Calcium (mg/L)	Without caries	16	173.81	52.93	-0.865	0.39	-0.301
	With caries	17	188.01	40.96			
Protein (g/L)	Without caries	16	4.65	1.58	2.213	0.04*	0.771
	Without caries	17	3.70	0.75			

SD = standard deviation, ¶ = Independent t-test, † = effect size, * = significance at *P* < 0.05

**Figure 1:**
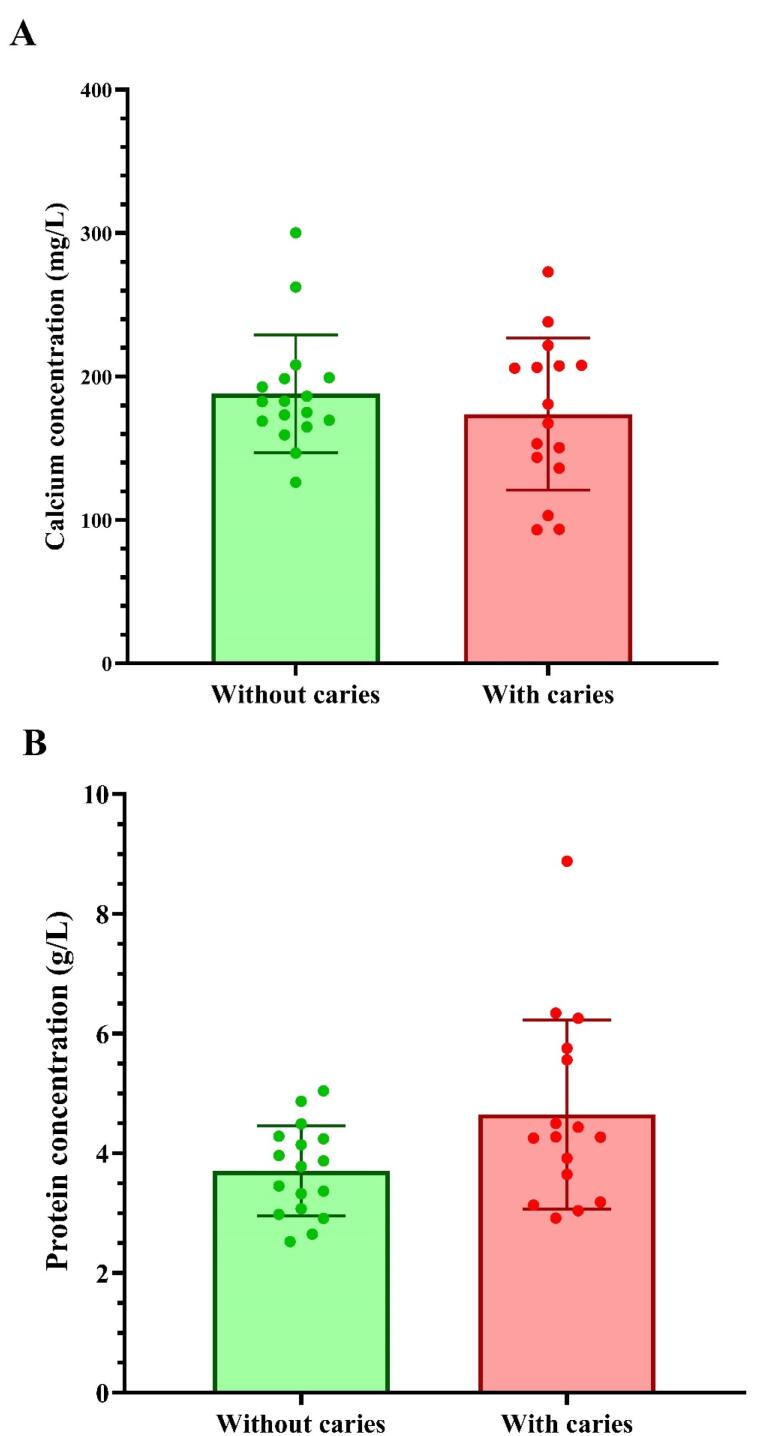
Distribution of calcium and protein concentrations in mothers’ breast milk for toddlers with and without caries. Each colored circle denotes a toddler. Error bars represent the standard deviation

Figure 1 illustrates the distribution of protein and calcium concentrations in two groups of toddlers—those with dental caries and those without. The mean protein concentration was significantly higher in the caries-free group compared to the group with dental caries (Figure 1B and Table 2). However, there was no significant difference in calcium concentration between the study groups (Figure 1A and Table 2); thus, the calcium concentration was relatively similar in participants.

 Binary logistic regression was employed to estimate the possible association between predictor variables (protein level, calcium level, mother’s age [in years], toddler’s age [in months], and toddler’s gender) and dental caries in toddlers. The initial model was developed using all toddlers (n = 33), revealing two cases identified as outliers (ZRE > |2| and COO > 0.7) that should be excluded from the model. Accordingly, the refined (final) model was established following outlier removal (n = 31). Table 3 presents these two models.

**Table 3:** Binary logistic regression models for predicting severe early childhood caries (S-ECC) in toddlers

**Predictors**	**β**	**SE**	**Wald**	**df**	**OR**	**95% CI for OR [Lower, Upper]**	***P*** **-value**
**Initial model (full sample [N=33])**							
Protein concentration	-0.999	0.528	3.581	1	0.368	[0.131, 1.036]	0.058
Calcium concentration	-0.002	0.010	0.059	1	0.998	[0.979, 1.017]	0.809
Mother’s age (year)	-0.062	0.121	0.262	1	0.940	[0.741, 1.192]	0.609
Toddler’s age (month)	0.101	0.210	0.231	1	1.106	[0.733, 1.670]	0.631
Toddler’s gender	1.362	0.847	2.587	1	3.905	[0.742, 20.539]	0.108
**Refined model (after outlier removal [N=31])**							
Protein concentration	-1.765	0.833	4.487	1	0.171	[0.033,.877]	0.034^*^
Calcium concentration	0.002	0.011	0.027	1	1.002	[0.980, 1.024]	0.870
Mother’s age (year)	-0.061	0.138	0.193	1	0.941	[0.718, 1.234]	0.660
Toddler’s age (month)	2.724	3.143	0.751	1	15.236	[0.032, 7216.228]	0.386
Toddler’s gender	2.696	1.247	4.672	1	14.818	[1.286, 170.782]	0.031^*^

95% CI = 95% confidence interval, OR = odds ratio, S.E. = standard error for regression coefficient, β = regression coefficient, * = significance at *P* < 0.05

 The refined model exhibited enhanced statistical properties while preserving practical classification accuracy. The notable improvement in model chi-squared significance (*P* = 0.011 vs. *P* = 0.117) and the increase in Nagelkerke R Square (0.508 vs. 0.313) suggest that excluding outliers led to a more robust and dependable predictive model.

 In the refined model, both protein concentration and the toddler’s gender were significant predictors of caries risk (*P* < 0.05). Increased protein levels were strongly associated with lower odds of caries (OR = 0.171), whereas being male was associated with a much higher risk—boys were about 14.8 times more likely than girls to develop caries. Meanwhile, calcium concentration, mother’s age, and toddler’s age did not show significant effects.

 Higher concentrations of protein in breast milk provide significant protection against dental caries, with each unit increase associated with an 82.9% reduction in the odds of developing caries (OR = 0.171, 95% CI: 0.033–0.877, *P* = 0.034). This finding supports the biological plausibility of the multiple protective mechanisms of breast milk proteins, suggesting that protein levels could be valuable for risk assessment and the development of targeted interventions.

## Discussion

 It appears that there are still unanswered questions regarding the etiology of S-ECC. For example, is there any association between the presence of S-ECC in toddlers and the levels of protein and calcium within mothers’ breast milk? In our study, the average amounts of calcium in breast milk were 188.01 g/mL (in the S-ECC group) and 173.81 g/mL (in the non-S-ECC group) (Table 2). Rios-Leyvraz and Yao^21^ reported that the calcium levels in human breast milk can range from 183 to 281 mg/L. It is higher in breast milk during the first days after the baby’s birth and decreases slightly as the baby grows older. In the current study, the children were 1‒3 years old, so the calcium content of their mothers’ breast milk was < 200 mg/L. No experimental studies are available on the effectiveness of breast milk calcium in preventing S-ECC; therefore, we were unable to compare our study with other studies. Only in vitro studies have shown that exposure of primary tooth enamel to breast milk can increase its surface calcium content, suggesting a potential protective effect.^22^ In the present study, there was no significant difference in breast milk calcium concentrations between the two groups.

 Proteins may decrease pH and trigger tooth demineralization.^23^ Research indicates that breast milk protein levels range from 8 to 10 g/L after 3‒4 months postpartum, with subsequent reductions observed thereafter.^24^ In the present study, the average amounts of breast milk protein among the mothers with children aged 12‒24 months were 3.70 g/L (the S-ECC group) and 4.65 g/L (the non-S-ECC group) (Table 2). If breast milk protein is insufficient, the baby may not be satisfied with sucking on the mother’s breast, so the baby will continue sucking for more times and/or for a longer duration. A couple of studies have reported that extended breastfeeding may result in higher lactose levels in milk; consequently, this increase in the frequency of breastfeeding and in lactose content can eventually lead to more caries.^25,26^

 It might also be stated that as the protein content of breast milk decreases after one year of age, the likelihood of dental caries increases with prolonged breastfeeding.^17^

 No research has been conducted on the protein content of mothers’ breast milk and its association with the incidence of S-ECC in their children’s dentition, so we were unable to compare our study with another. Figure 1 shows the average protein concentration in two groups of toddlers with and without dental caries. According to this chart, the average protein concentration in caries-free toddlers was significantly higher than in those with dental caries. This indicates that protein intake in toddlers under 24 months may positively influence the reduction of both the extent and severity of dental caries, as also predicted by our refined regression model. The results coincide with those of an investigation by Hooley et al.,^27^ which demonstrated that the average protein concentration in schoolchildren without caries was significantly higher than in those with dental caries.

 The study was limited by a small sample size and by certain mothers’ reluctance to provide their milk samples. Male children exhibited a significantly greater risk of S-ECC compared to female toddlers (OR = 14.818, 95% CI: 1.286–170.782, *P* = 0.031). Although the wide confidence interval (CI) reflects statistical uncertainty due to the small sample size, these findings highlight the importance of considering gender differences in preventive strategies and underscore the need for further research in larger populations. Although the refined model reached statistical significance, its classification accuracy of 64.5% suggests that a substantial portion of the variance in caries status remains unaccounted for. Additionally, our study did not explore the specific behavioral, hygiene, and social factors that may mediate gender differences in caries risk. The association between lactose and S-ECC in 12‒24-month-old toddlers has been addressed in a prior publication by the authors.^26^ Hence, this underscores the complex, multifactorial nature of caries development and highlights the need to include additional behavioral, environmental, and microbiological variables in more comprehensive predictive models.

## Conclusion

 Overall, our findings indicate that a higher protein intake from breastfeeding in toddlers under 24 months, combined with being female, is linked with a lower risk of S-ECC. Our information can be beneficial for developing targeted preventive interventions in pediatric dentistry. Interventions may emphasize promoting optimal breastfeeding practices and educating parents about the significance of nutrition in early childhood dental health. Further research may identify additional factors contributing to the risk of S-ECC, facilitating the development of more comprehensive prevention strategies.

## Competing Interests

 The authors declare that they have no conflicts of interest.

## Data Availability

 The data that support the findings of this study are available from the corresponding author upon reasonable request.

## Ethical Approval

 The Ethics Committee of Kerman University of Medical Sciences granted ethical approval for this research (IR.KMU.REC.1403.358). Also, informed consent was attained from mothers.
